# Aggressive variant prostate cancer with multiple subcutaneous metastases: a case report

**DOI:** 10.1007/s13691-024-00673-7

**Published:** 2024-04-18

**Authors:** Yusuke Hoshino, Kent Kanao, Yu Miyama, Takeo Kosaka, Go Kaneko, Suguru Shirotake, Masanori Yasuda, Masafumi Oyama

**Affiliations:** 1 Department of Uro-Oncology, Saitama Medical University International Medical Center, 1397-1 Yamane, Hidaka, Saitama, 350-1298 Japan; 2 Department of Pathology, Saitama Medical University International Medical Center, 1397-1 Yamane, Hidaka, Saitama, 350-1298 Japan; 3 Department of Urology, Keio University School of Medicine, 35 Shinanomachi, Shinjuku-ku, Tokyo, 160-8582 Japan

**Keywords:** Aggressive variant prostate cancer, Neuroendocrine differentiation, Neuroendocrine prostate cancer, Subcutaneous metastasis

## Abstract

A 71-year-old man with bone metastasis of hormone-sensitive prostate cancer was treated with androgen deprivation therapy and apalutamide. Radium-223 and radiation therapy were administered after it become castration resistant. Although prostate-specific antigen levels remained low, multiple subcutaneous metastases of neuroendocrine prostate cancer were observed. A review of the pre-treatment prostate needle biopsy revealed a small component with features suggestive of neuroendocrine differentiation. Phosphatase and tensine homolog loss and tumor protein p53 overexpression were observed, confirming the diagnosis of aggressive variant prostate cancer. Platinum-based chemotherapy was administered; however, the patient died 28 months after diagnosis. In this case, if the diagnosis of aggressive variant prostate cancer had been made at an earlier time by biopsy specimens, there might have been a possibility to improve the prognosis by the earlier introduction of the platinum-based regimen.

**Supplementary Information:**

The online version contains supplementary material available at 10.1007/s13691-024-00673-7.

## Introduction

Neuroendocrine prostate cancer (NEPC) is a histological subtype of prostate cancer known for its aggressiveness, unfavorable clinical outcomes, and expression of neuroendocrine markers. De novo NEPC is rare and typically develops after hormonal therapy, which is referred to as treatment-related neuroendocrine prostate cancer (t-NEPC) [1], 1].

The frequency of t-NEPC has increased, particularly with the introduction of novel androgen receptor pathway inhibitors (ARPIs), raising clinical concerns. In an autopsy study, the frequency of prostate cancer with AR-negative with neuroendocrine features was 6.3% before the introduction of ARPIs, but has increased to 13.3% since their introduction [3]. The current NCCN guidelines recommend platinum-based regimens as the first line and subsequent treatment for t-NEPC [4].

However, diagnosing NEPC histologically can be challenging in some patients. Therefore, a phenotypic syndrome called aggressive variant prostate cancer (AVPC), which includes poor-risk features, has been defined using criteria that do not require the mandatory diagnosis of NEPC. Several definitions of AVPC have been proposed in clinical trials. A recent phase 2 study demonstrated that cabazitaxel plus carboplatin improves the median progression-free survival for men with AVPC which was defined as meeting any of seven criteria, including unfavorable genomics (involving defects in at least two of phosphatase and tensine homolog (PTEN), tumor protein p53 (TP53), and retinoblastoma 1 (RB1)) [5].

Therefore, early diagnosis of AVPC and the use of platinum-based regimens are essential for improving the prognosis of AVPC. However, there are few case reports, and clinicians are not sufficiently informed about the clinical presentation.

Herein, we report a case of AVPC with multiple subcutaneous metastases. We also summarized all previous case reports of AVPC (Table 1). Furthermore, we compare the immunohistochemical expression of PTEN, TP53, and RB1 between biopsy specimens at diagnosis and subcutaneous metastases to investigate the utility of immunohistochemical staining for early diagnosis of AVPC.

**Table 1:** Literature review of AVPC cases

Author	Age	PSA at initial diagnosis (ng/ml)	Gleason score at initial diagnosis	Stage at initial diagnosis	Previous treatment	Interval between initial and AVPC diagnosis (months)	PSA at AVPC diagnosis (ng/ml)	Metastatic site at AVPC diagnosis	Gene mutations	Subsequent treatment	Outcome
Our case	71	86.89	4 + 5 = 9	T3N1M1b	ADT + APA Ra223 EBRT	26	0.112	Subcutaneous Lymph nodes Bone	*RB1* loss, *TP53* mutation (IHC)	EP	Dead in 2 months
Weng et al.[8]	77	6.78	4 + 4 = 8	T4N0M1b	CAB Abiraterone Docetaxel	55	3.27	Brain Lung Bone	*AR, EXT1, MYC, BRAF* mutations (genetic test)	–	Dead
Masuda et al.[9]	79	15.54	5 + 5 = 10	N + M0	CAB	41	n. d	Urethra	LOH of *BRCA2*, amplification of *KRAS* (targeted next-generation sequencing)	TUR, EP	BSC

*ADT* androgen deprivation therapy, *APA* apalutamide, *AR* androgen receptor, *BRAF* v-raf murine sarcoma viral oncogene homolog B, *BRCA* breast cancer gene 1, *BSC* best supportive care, *CAB* combined androgen blockade, *EBRT* external beam radiation therapy, EP cisplatin plus etoposide, *EXT1* Exostosin-1, *IHC* immunohistochemistry, *KRAS* Kirsten rat sarcoma virus, LOH loss of heterozygosity, *MYC* myelocytomatosis, n. d. no data, *PSA* prostate-specific antigen, *RB1* retinoblastoma 1, *TP53* tumor protein p53, *TUR* transurethral resection

## Case report

A 71-year-old Japanese man presented with elevated serum prostate-specific antigen (PSA) (86.89 ng/ml). He was asymptomatic; however, a hard, stony nodule was found in the left lobe of his prostate gland on digital rectal examination. Transrectal ultrasound-guided prostate needle biopsy revealed 12 cores (out of 12) of adenocarcinoma with a Gleason score of 4 + 5 = 9 and grade group 5. There were some components of intraductal carcinoma of the prostate (IDC-P). A whole-body examination revealed seminal vesicle invasion of the prostate tumor, left iliac and obturator lymph node metastases, and iliac and pubic bone metastases. Based on these results, the disease stage was cT3bN1M1b, low-risk (LATITUDE definition [6]), and low-volume (CHAARTED definition [7]).

**Fig. 1:**
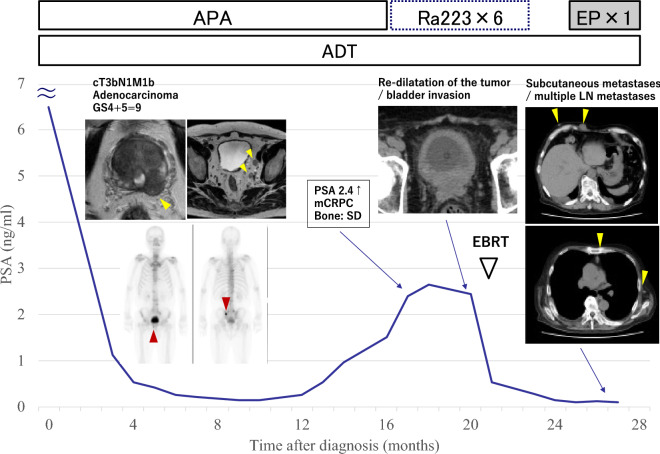
Serum PSA level and time course of treatment

Androgen deprivation therapy (ADT) with degarelix acetate and apalutamide (1000 mg/day) was initiated. PSA reached a nadir of 0.152 ng/mL in 9 months but increased to 2.4 ng/mL 16 months later. Lymph node metastasis reduced and no visceral metastases appeared at that time, although bone metastasis persisted. He was, then, diagnosed with castration-resistant prostate cancer (CRPC) and administered radium-223 because PSA doubling time was relatively slow. His serum PSA level dropped to < 0.2 ng/mL immediately; however, hematuria, urinary retention, and pelvic pain appeared during the course of treatment. Computed tomography (CT) revealed re-dilatation of the prostate tumor, bladder invasion, and left external iliac lymph node metastasis. He underwent salvage external beam radiation therapy (EBRT) of 39 Gray delivered in 13 fractions for the prostate tumor and bladder invasion. Six courses of radium-223 were administered in total. The PSA level remained low (0), and only ADT was continued. Twenty-six months after diagnosis, multiple painless subcutaneous nodules began to appear, primarily on the chest. On imaging, multiple lymph node metastases were recognized in the axillary, mediastinal, and para-aortic regions as well as new metastases in the spine. Rapid progression of the prostate cancer occurred, although the patient’s serum PSA level was only 0.112 ng/mL (Fig. 1).

**Fig. 2:**
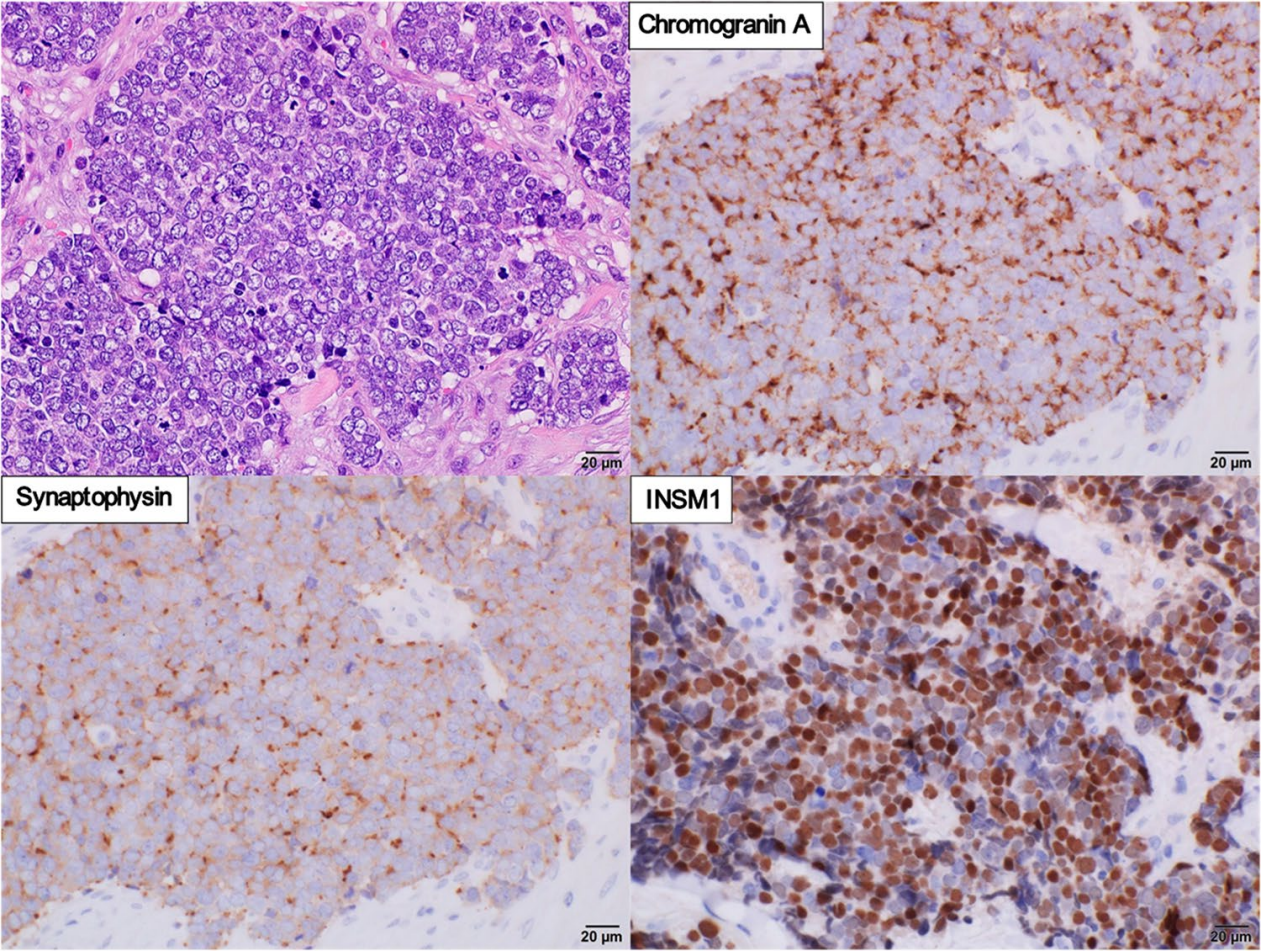
Pathological findings of metastatic prostatic small cell carcinoma. The tumor cells with high nuclear-to-cytoplasmic ratio form sheet-like and solid structures. Immunohistochemically, the tumor cells are diffusely positive for chromogranin A, synaptophysin, and INSM1

The subcutaneous nodule consisted of tumor cells with a high nuclear-to-cytoplasmic ratio, which differed from that of the adenocarcinoma. Immunostaining was negative for PSA and androgen receptor (AR) and positive for synaptophysin, chromogranin A, and insulinoma-associated 1 (INSM1) (Fig. 2). Blood tests revealed high levels of neuron-specific enolase (NSE) (1480 ng/mL) and pro gastrin releasing peptide (ProGRP) (557 ng/mL), suggesting neuroendocrine prostate cancer (NEPC).

**Fig. 3:**
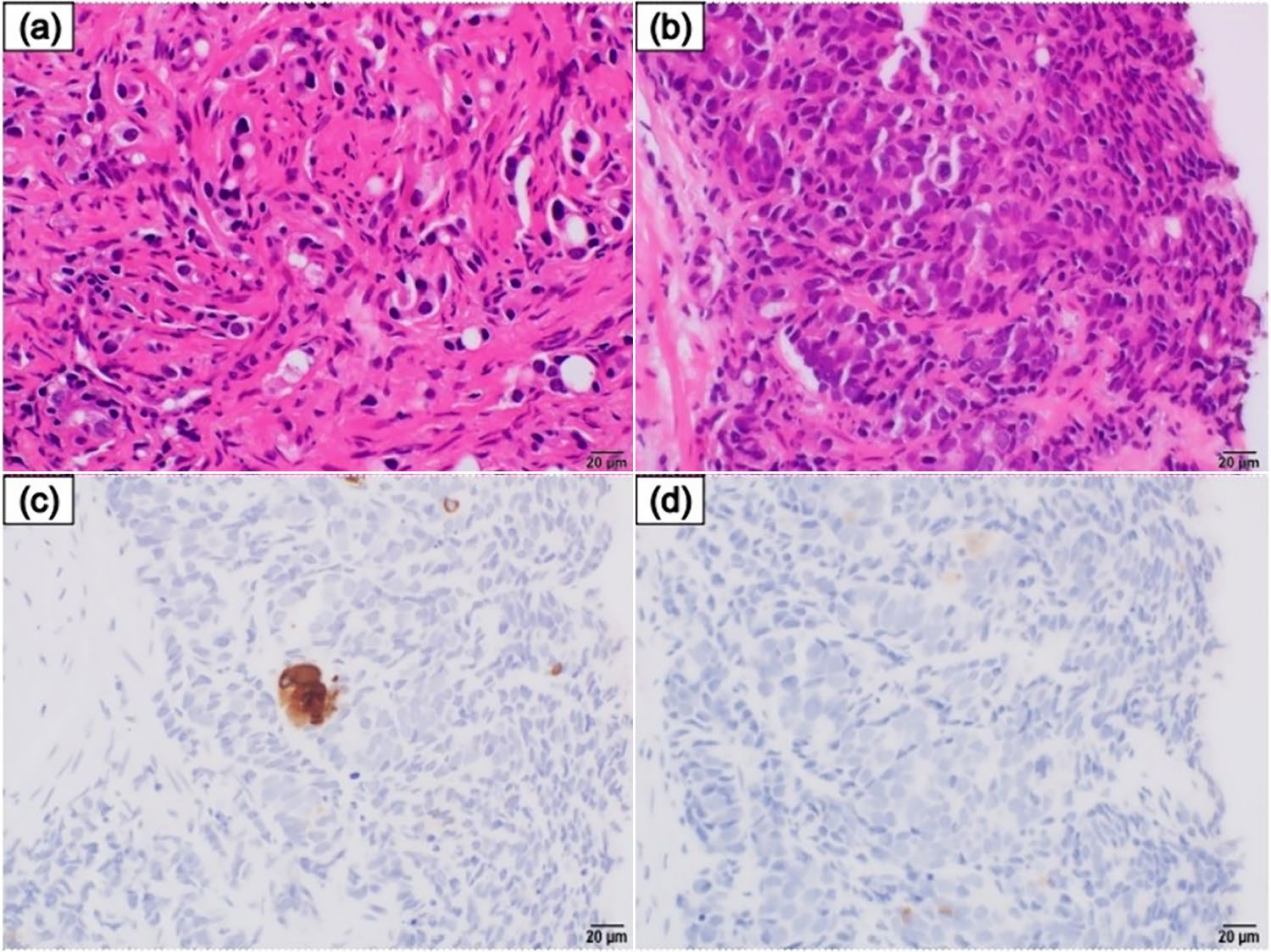
Pathological findings of primary prostatic acinar adenocarcinoma. **a** The tumor cells with swelling round nuclei and eosinophilic cytoplasm form vaguely acinar and trabecular structures. Solitary cells are also seen. We classified this area as Gleason pattern 4 + 5 and grade group 5. **b** Focally, the tumor cells with high nuclear-to-cytoplasmic ratio form a compact nest. Retrospectively, we considered the possibility of neuroendocrine differentiation. However, chromogranin A (**c**) and synaptophysin (**d**) expression was very focal. Therefore, neuroendocrine differentiation was unclear and we classified this area as Gleason pattern 4 + 4 and grade group 4

The pre-treatment prostate biopsy specimens were reviewed (Fig. 3). Although normal acinar adenocarcinoma was predominant, one specimen from the medial left apex, diagnosed with Gleason score of 4 + 4 = 8 and grade group 4, consisted of tumor cells with a high nuclear-to-cytoplasmic ratio that formed a compact nest. Immunostaining was negative for PSA and INSM1, but positive for synaptophysin and chromogranin A. It resembled a metastatic tumor and contained components suggestive of neuroendocrine differentiation (NED). Cisplatin plus etoposide (EP) was administered; however, the metastases enlarged on CT one month later, and the patient’s general condition deteriorated. He underwent only one course of therapy and died 28 months after diagnosis.

## Discussion

Recently, rapidly progressing and highly malignant prostate cancer have been phenotypically defined as AVPC. Clinically, AVPC is characterized by distinctive manifestations, such as predominantly visceral or lytic bone metastases and the presence of bulky tumor masses, frequently in the setting of low PSA level with high-volume tumor burden and early emergence of castration resistance [8]. While several clinical trials on AVPC have been reported, to our knowledge, there have been only two cases of AVPC documented in the English literature (Table 1) [9, 10].

The current NCCN guidelines define AVPC as meeting either of seven criteria: visceral metastases, low PSA and bulky disease, high carcinoembryonic antigen (CEA), high lactate dehydrogenase (LDH), lytic bone metastases, NEPC histology or unfavorable genomics (defects in at least two of PTEN, TP53, and RB1) [4]. The guidelines indicate that cabazitaxel plus carboplatin with growth factor support can be considered for fit patients with AVPC based on a recent phase 2 study [5]. Through referring to the NCCN criteria, this case had high LDH (1482 U/L when subcutaneous metastases were observed) and t-NEPC histology.

In this report, we compared biopsy specimen and subcutaneous metastases tissue immunobiologically to investigate whether the diagnosis of NEPC or AVPC was possible at the biopsy. Table 2 presents the results of the comparison. There are tumor cells with high nuclear-to-cytoplasmic ratio form a compact nest focally in a biopsy specimen. The morphology is suggestive of NED, and immunostaining was negative for PSA and INSM1 but positive for synaptophysin and chromogranin A. RB1 loss and TP53 overexpression were observed in NEPC metastases, but the NED-suspected component did not show variants of PTEN, TP53, or RB1. Conversely, PTEN loss and TP53 overexpression were observed in acinar adenocarcinoma (classified as Gleason score 4 + 4 = 8), confirming the nature of AVPC (overexpression was defined as a mutation of TP53 > 10% [11]). We did not regard this case as de novo NEPC. While the presence of chromogranin A and synaptophysin expression suggests NED, it does not necessarily mean NEPC; immunohistochemical analysis of metastatic hormone-sensitive prostate cancer reveals that 61% of primary cancer specimens are positive for chromogranin A [12]. Furthermore, the NED-suspected component was focal and the variants of PTEN, TP53, or RB1 were negative, which were different from those observed in NEPC metastases. Hence, we could not conclude that the NED-suspected component had progressed through the treatment to become NEPC portion. It would be overstated to classify this component as de novo NEPC. The term t-NEPC would likely be more appropriate.

**Table 2:** Expression of tumor-suppressor genes and AR in primary (acinar adenocarcinoma and NED) and metastatic lesions

	Primary lesion		Metastatic lesion
	acinar	NED	Small cell carcinoma/NEPC
PTEN	− /loss	+ /retained	+ /retained
TP53	overexpression +	overexpression −	overexpression +
RB1	+ /retained	+ /retained	− /loss
AR	+ /retained	+ /retained	− /loss

*AR* androgen receptor, *NED* neuroendocrine differentiation, *NEPC* neuroendocrine prostate cancer, *PTEN* phosphatase and tensine homolog, *RB1* retinoblastoma 1, *TP53* tumor protein p53

Currently, as the clinical significance remains uncertain, it is not recommended to routinely use immunohistochemistry stains to detect any NED in an otherwise morphologically typical primary adenocarcinoma of the prostate [13]. However, for the early introduction of platinum-based regimens for NEPC and AVPC, it may be useful to diagnose NED and perform immunostaining for PTEN, TP53, and RB1 in biopsy specimens, and we await the accumulation of future research results. In addition, comprehensive genomic profiling (CGP) testing was not performed at the time when this case progressed to CRPC after apalutamide administration. Through genomic testing, if genetic alterations of PTEN, TP53, and RB1 were found, it might have been possible to diagnose AVPC, and platinum-based regimens could have been introduced before initiating radium-223. However, it is not standard medical practice to conduct CGP testing at this point under the current Japanese insurance system. This case might suggest that early recognition of AVPC could be achieved by immunohistochemical examination of CRPC tissue instead of CGP testing.

We confirmed the diagnosis of NEPC through histological examination of the metastatic subcutaneous nodules. Subcutaneous metastasis of prostate cancer is extremely rare, accounting for only 0.09% of all prostate cancers [14]. In this case, we prioritize lymphogenous metastasis over hematogenous or other types due to the localization of subcutaneous metastases on the chest, and the identification of multiple lymph node metastases in the axillary and mediastinal regions.

In conclusion, understanding the definition of AVPC and utilizing genomic markers like PTEN, TP53, and RB1 may offer the potential to improve prognosis by early introduction of the platinum-based regimens.

### Supplementary Information

Below is the link to the electronic supplementary material.

Supplementary file1 PTEN, TP53, RB1 and AR expression in primary acinar adenocarcinoma. (**a**) PTEN expression was focally lost. (**b**) TP53 exhibited overexpression in some area. (**c**) RB1 expression was retained. (**d**) AR expression was retained (DOCX 151 KB)

Supplementary file2 PTEN, TP53, RB1 and AR expression in metastatic NEPC. (**a**) PTEN expression was retained. (**b**) TP53 exhibited overexpression. (c) RB1 expression was lost. (**d**) AR expression was lost (DOCX 125 KB)

## Data Availability

The data that support the findings of this study are available from the corresponding author upon reasonable request.
