# Supplementary figures and images for: Aggressive variant prostate cancer with multiple subcutaneous metastases: a case report
Source: Int Cancer Conf J. 2024 Apr 18;13(3):250–5. doi: 10.1007/s13691-024-00673-7 (PMC11217196; doi:10.1007/s13691-024-00673-7)

**Supplementary Fig. 1**


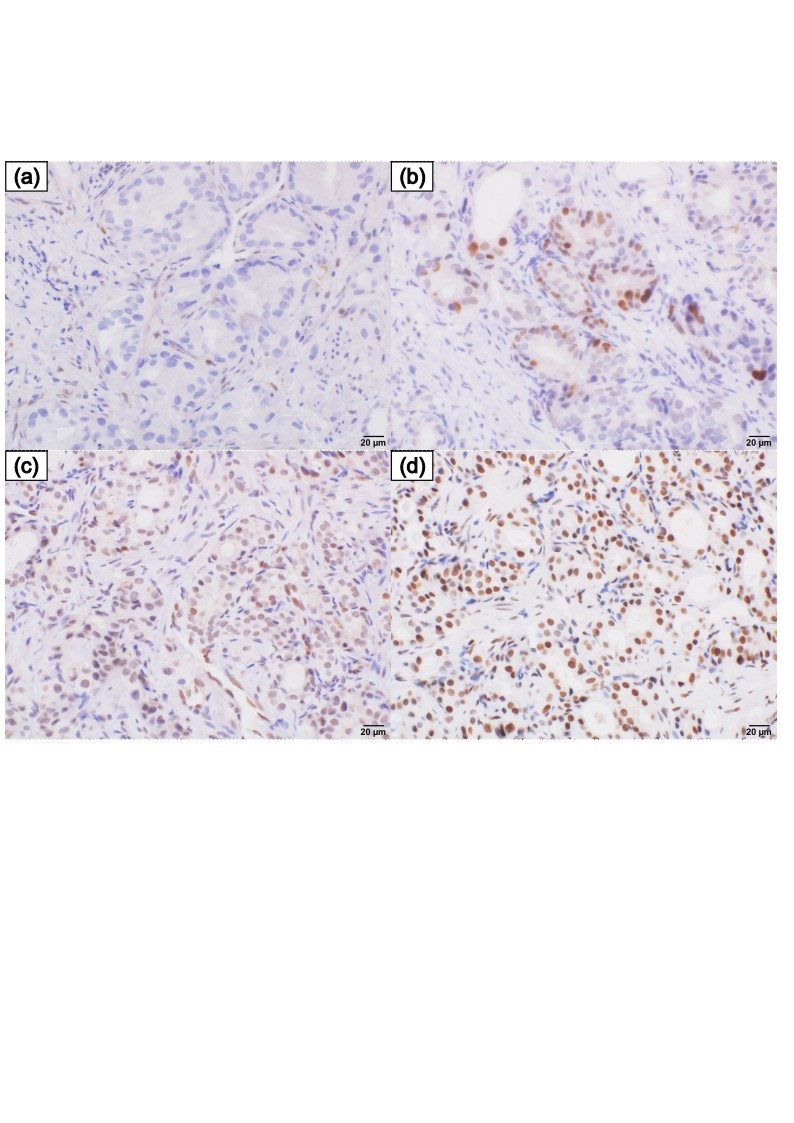

Supplement: Supplementary file 1 — Supplementary file1 PTEN, TP53, RB1 and AR expression in primary acinar adenocarcinoma. (a) PTEN expression was focally lost. (b) TP53 exhibited overexpression in some area. (c) RB1 expression was retained. (d) AR expression was retained (DOCX 151 KB) [file 13691_2024_673_MOESM1_ESM.docx]

**Supplementary Fig. 2**


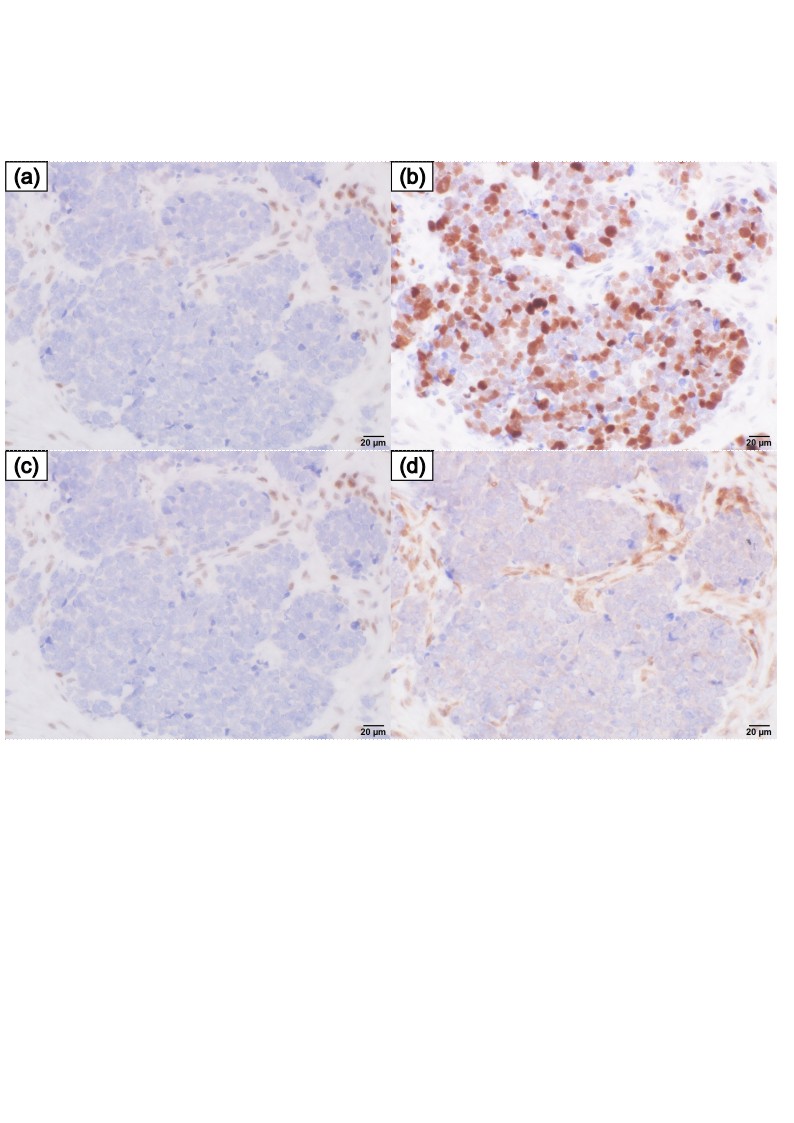

Supplement: Supplementary file 2 — Supplementary file2 PTEN, TP53, RB1 and AR expression in metastatic NEPC. (a) PTEN expression was retained. (b) TP53 exhibited overexpression. (c) RB1 expression was lost. (d) AR expression was lost (DOCX 125 KB) [file 13691_2024_673_MOESM2_ESM.docx]
